# Evaluation of cardiomyopathy diagnosis in heart transplant recipients: comparison of echocardiographic and pathologic classification

**DOI:** 10.1186/s43044-021-00154-9

**Published:** 2021-03-25

**Authors:** Neda Behzadnia, Babak Sharif-Kashani, Zargham Hossein Ahmadi, Farah Naghashzadeh, Atosa Dorudinia, Alireza Jahangirifard, Hamoun Hamarz, Payam Abbasi

**Affiliations:** 1 Lung Transplantation Research Center, National Research Institute of Tuberculosis and Lung Diseases (NRITLD), Shahid Beheshti University of Medical Sciences, Tehran, Iran; 2 Chronic Respiratory Diseases Research Center, National Research Institute of Tuberculosis and Lung Diseases (NRITLD), Shahid Beheshti University of Medical Sciences, Tehran, Iran; 3 Tracheal Diseases Research Center, National Research Institute of Tuberculosis and Lung Diseases (NRITLD), Shahid Beheshti University of Medical Sciences, Tehran, Iran; 4 Nikan Education and Research Center, Tehran, Iran

## Abstract

**Background:**

Definite diagnosis of cardiomyopathy types can be challenging in end-stage disease process. New growing data have suggested that there is inconsistency between echocardiography and pathology in defining type of cardiomyopathy before and after heart transplantation. The aim of the present study was to compare the pre-heart transplant echocardiographic diagnosis of cardiomyopathy with the results of post-transplant pathologic diagnosis.

**Results:**

In this retrospective cross-sectional clinicopathological study, 100 consecutive patients have undergone heart transplantation in Masih-Daneshvari hospital, Tehran, Iran, between 2010 and 2019. The mean age of patients was 40 ± 13 years and 79% of patients were male. The frequency of different types of cardiomyopathy was significantly different between two diagnostic tools (echocardiography versus pathology, *P* < 0.001). On the other hand, in 24 patients, the results of echocardiography as regard to the type of cardiomyopathy were inconsistent with pathologic findings.

**Conclusion:**

Based on the findings of the present study, it could be concluded that there is a significant difference between echocardiographic and pathologic diagnosis of cardiomyopathy; therefore, it is necessary to use additional tools for definite diagnosis of cardiomyopathy like advanced cardiac imaging or even endomyocardial biopsy before heart transplantation to reach an appropriate treatment strategy.

## Background

Heart transplantation (HT) is a surgery in which a donor heart is transplanted to a selected recipient with advanced refractory heart failure. HT is referred as the final treatment strategy of end-stage heart failure (HF) in some cases, when none of the medical therapies are effective [1].

As well, cardiomyopathy (CMP) is a disease of the heart muscle that directly affects and also multiple etiologic factors might be contributing, the most common are coronary artery disease, poorly controlled hypertension, toxins (alcohol, chemotherapeutic agents with cardiotoxicity), valve diseases, infections (viral myocarditis, Chagas cardiomyopathy), inflammatory diseases, and even genetic alterations (hypertrophic cardiomyopathy, family history of dilated cardiomyopathies, arrhythmogenic right ventricular dysplasia, etc.) [2].

According to the WHO classification for cardiomyopathies, this disease is divided morphologically into dilated, hypertrophic, and restrictive types. The most common type is dilated and its prevalence reaches to 36.5 in 100,000 population, while the restrictive type is the least common [3, 4]. The mortality of cardiomyopathy is high; 5 and 10 years survival is 50% and 25%, respectively [4, 5].

The prevalence of heart failure is increasing and one of the most important causes is coronary artery disease [6, 7]. However, many advances have been developed in the treatment of patients with heart failure, these advances have a positive impact on patients’ survival and quality of life [8]. Nevertheless, the standard final treatment for these patients is heart transplantation [9]. Despite extensive evaluation of cardiomyopathy clinical diagnosis, a definite type of CMP remains unknown in 40% to 50% of cases before cardiac transplantation and is therefore diagnosed as idiopathic dilated cardiomyopathy (IDC) [10, 11]. As well, evaluation of pathologic results of heart failure patients may help to accurate diagnosis of cardiomyopathy; also, its comparison with the initial clinical diagnosis can also be examined [10, 12].

Echocardiography, cardiac magnetic resonance imaging (CMR), and endomyocardial biopsy are known as choice diagnostic methods used for diagnosis and assessment of various cardiac diseases like myocarditis and some forms of infiltrative cardiomyopathies. Each of these methods has its advantages and drawbacks and their efficacy varies according to different cardiac diseases [13, 14]. However, in the past years, advances and sophistication in advanced methods of echocardiography and cardiac magnetic resonance imaging (CMR) introduced more application of imaging techniques than endomyocardial biopsy (EMB) [15].

Up to the present time, no accurate diagnostic algorithm has been developed for definite diagnosis of cardiomyopathy [15], and therefore, the pathologic results are inconsistent with echocardiographic diagnosis. Since accurate etiologic diagnosis of cardiomyopathy before heart transplantation may alter treatment strategy and in some cases like myocarditis or inflammatory cardiomyopathies provide a chance of recovery with specific treatment and better survival and even eliminate the need to transplant, therefore, by designing this study, we aimed to assess accuracy of clinical cardiomyopathy diagnosis mainly by echocardiography with anatomopathological findings (gold standard) of explanted hearts*.*

## Methods

### Study population

In this retrospective cross sectional study, a total of 100 candidates for heart transplantation who admitted to the heart transplant department of Masih Daneshvari hospital, Tehran, Iran, in 2010–2019, were selected. All heart transplant candidates referred from other hospitals around the country to our center and had previous multiple hospitalization in years before candidacy and had refractory heart failure symptoms with maximal pharmacological and guideline-directed device therapy and had a complete assessment by experienced heart transplant team with appropriate pretransplant tests and imaging studies (echocardiography, cardiac CT, CMR, cardiac cath and coronary angiography) as indicated. All patients that were not bedridden and inotropic-dependent in the hospital did cardiopulmonary exercise testing, and in no eligible case, we had VO2 max more than 10 ml/kg/min. Demographic, clinical, and paraclinical data of patients with focus on the diagnosis of cardiomyopathy before (based on echocardiographic findings and clinical data) and after (based on pathologic findings of the explanted heart) heart transplantation were collected from electronic medical records and was reviewed by two experienced cardiologists of the heart transplant team and pretransplant clinical diagnosis was made by them. Post-transplant anatomopathological diagnosis was performed by a cardiovascular pathologist who was unaware of clinical diagnosis and consisted of macroscopic and microscopic study. The explanted hearts were fixed in buffered formalin. Standard samples were taken from the two ventricles (free walls), septum, coronary vessels, and valvular system. Histological 5-micron-thick sections and routine staining (hematoxylin and eosin, Masson’s trichrome) were performed. In selected cases, special dyes (e.g. Congo red and thioflavin in patients with suspected amyloidosis) and inmumohistochemical techniques (e.g. viral myocarditis) were employed. The histological diagnosis of myocarditis was made according to the Dallas criteria. The study protocol was approved by the Ethical Committee of Shahid Beheshti University of Medical Sciences (Committee’s reference number: IR.SBMU.NRITLD.REC.1396.398) and informed consent was obtained from all participants before entering the study.

### Statistical analysis

The software SPSS 25 package (SPSS Inc., Chicago, IL) was used for statistical calculations and data analysis. Data are expressed as mean ± standard deviation (SD). The normality of data was assessed by Kolmogorov-Smirnov *Z*-test. As well as this, *χ*^2^ test was used for testing relationships between categorical variables. Statistical significance was considered when the *P* value was < 0.05.

## Results

### Description of patients

The mean age of patients was 40 ± 13 years and 79% of patients were male. Ninety-five percent of patients had normal sinus rhythm in ECG and 49% showed an LBBB pattern. We observed that 38% of individuals had intracardiac devices (36% implantable cardioverter defibrillator (ICD)/2% cardiac resynchronization therapy (CRT)) and the mean weight of explanted heart was 438 ± 130 g. Positive history of coronary artery bypass surgery (CABG), percutaneous coronary intervention (PCI) with coronary stents, and heart valve replacement was seen in 5, 6, and 4% of patients, respectively. All the patients had complete detailed transthoracic echocardiography before transplant often multiple by an experienced echocardiologist and transesophageal echocardiography as needed. In echocardiography mean pretransplant LVEF in our cases was 12.27% and 100% showed evidence of RV systolic dysfunction according to 2015 ASE guidelines for cardiac chamber quantification by echocardiography in adults [16]. We detected moderate/severe functional tricuspid regurgitation (TR) in 76% and moderate/severe functional mitral regurgitation (MR) in 87% of our cases suggestive for advanced stage CMP process. Four patients had previous valve replacement in remote years with no evidence of prosthetic valve malfunction and there was a mean time window 6.5 years between cardiac surgery and advanced CMP and transplant. Mild to moderate pericardial effusion was seen in 14% of recipients. Serological and viral assay was done according to cardiac transplant protocol before allocation to surgery. All cases had done cardiac cath for hemodynamic study and if indicated coronary angiography, SPECT myocardial perfusion imaging, and cardiac CT scan. As cardiac magnetic resonance (CMR) imaging was not accessible in our center, CMR performed in a few cases suspicious to myocarditis or infiltrative diseases before transplant. Genetic assay was not easily available in our country and endomyocardial biopsy was performed in only 5 cases. Mean mortality after heart transplantation was 35%. Clinical characteristics of the patients and echocardiographic findingsare summarized in Tables 1 and 2.

**Table 1:** Demographic and clinical features of patients

Parameter	*N* (%)
Age^a^	40 (±13)
Gender, male^b^	79 (79)
History of CABG^b^	5 (5)
History of PCI^b^	6 (6)
History of heart valve replacement^b^	4 (4)
Type of heart rhythm^b^	
NSR	95 (95)
AF	5 (5)
ECG findings^b^	
Q wave or old MI	29 (29)
LBBB	49 (49)
LVH	22 (22)
Intracardiac device^b^	
ICD	36 (36)
CRT	2 (2)
Mortality^b^	35 (35%)
Cardiomyopathic heart weight (g)^a^	438 (± 130)

^a^Data expressed as mean (standard deviation)

^b^Data expressed as number (% of total)

**Table 2:** Echocardiographic findings of cardiomyopathic hearts

Parameters (%)	Normal	Mild	Moderate	Severe
Mitral regurgitation	0	14	57	30
Tricuspid regurgitation	10	14	34	42
Aortic insufficiency	67	33	0	0
Aortic stenosis	100	0	0	0
Pericardial effusion	86	7	7	0
Mean ejection fraction (EF%) (SD) ^a^RV systolic dysfunction(%)		12.27 (4.4) 100		

^a^RV (right ventricle) systolic dysfunction was defined quantitatively by echocardiographic-derived variable: TAPSE (tricuspid annular plane systolic excursion) and tissue-Doppler-derived tricuspid annulus systolic (*S*) velocity and myocardial performanace index (MPI) and fractional area change (FAC) according to 2015 ASE-guidelines for cardiac chamber quantification by echocardiography in adults and RV systolic dysfunction was defined with TAPSE < 17 mm, *S* velocity < 9.5 cm/s, MPI > 0.54 and FAC < 35%

### Comparison of pre-heart transplantation with post-heart transplant cardiomyopathy diagnosis

As is shown in Table 3, the frequency of different types of cardiomyopathy (CMP) was significantly different between two diagnostic tools (echocardiography versus pathology, *P* < 0.001). In 24 patients, the results of echocardiography as regard to the type of cardiomyopathy were not a match with pathologic findings. The frequency of different types of cardiomyopathy including DCM, ischemic CMP, valvular CMP, amyloidosis, hypertrophic CMP, and myocarditis in echocardiography assessment was 69, 24, 1, 0, 1, and 5%, respectively. This frequency in pathologic assessment was 57, 27, 4, 1, 1, and 10%, individually (Table 3).

**Table 3:** Comparison the frequency of different types of cardiomyopathy based on two different diagnostic methods

Type of cardiomyopathy	Echocardiography diagnosis	Pathology diagnosis	*P* value
DCM	69	57	< 0.001
Ischemic CMP	24	27	
Valvular CMP	1	4	
Amyloidosis	0	1	
Hypertrophic CMP	1	1	
Myocarditis	5	10	

Twelve cases that clinically diagnosed DCM regarding echocardiographic data after pathologic evaluation reclassified in other groups. The most discrepancy between echo versus pathology was observed in myocarditis category that we know DCM is the final consequence of chronic unresolved myocarditis and highlights the importance of pretransplant endomyocardial biopsy and CMR or additional serologic assays for better etiologic classification. All patients that had a remote history of surgical heart valve replacement reclassified in valvular CMP by pathologic diagnosis though their echocardiographic diagnosis was compatible with DCM.

## Discussion

Obviously, cardiomyopathy causes low cardiac output and/or elevated intracardiac pressures at rest or during stress that lead to heart failure. Heart transplantation is an accepted treatment for patients who are in the end-stage of heart failure [17]. Apart from serious early and long-term complications of heart transplantation, one of the most important challenges in this group of patients is a definite diagnosis of cardiomyopathy. Because, accurate diagnosis allows for proper treatment before transplantation and improves the patient outcome and in some familial genetically inherited states helps in family screening and detecting carriers or asymptomatic siblings [10, 18]. The aim of the present study was to provide an evidence-based document in order to compare the efficacy of two diagnostic tools (echocardiography versus pathology) in diagnosis of different types of cardiomyopathy in 100 patients who were underwent heart transplantation. Interestingly, we observed that pathologic assessment of cardiomyopathy is more reliable than echocardiography.

Our findings are in accordance with previous studies [10, 12, 19]. Similar to us, Angelini et al. [19] studied the discrepancy between clinical and pathological diagnosis in 257 patients who had undergone cardiac transplantation. They showed a discrepancy between clinical and pathological diagnosis in 8% of patients which is consistent with our findings in the present study. Huang et al. [12] carried out a study in 40 cardiac transplant recipients who had undergone cardiac transplantation to evaluate the discrepancy between pre- and post-transplant diagnosis of end-stage dilated cardiomyopathy and reported that post-transplant coronary heart disease diagnosis is significantly higher than that of pre-transplant. The results of the study done by Huang et al. are completely in accordance with the present study data. One more similar study by Iván Constantin et al. reported a significant agreement between pre-transplant diagnosis and pathological anatomy [20]. In approximately 1 out of every 4 patients, the echocardiographic diagnosis did not match with pathology. This discrepancy may be in part due to the fact that in more than half of IDC cases the diagnosis was made in the explanted heart. These results are similar to those reported by Luk et al. [10]. One of the main reasons of a discrepancy between echocardiography and pathology in our study was related to valvular CMP. The point is how the clinician performed the classification based on echocardiographic data, if the patients with previous valvular surgery were clinically reclassified as valvular CMP this discrepancy would be reduced. This issue should be considered in future etiologic classification of CMP in heart transplant recipients.

Etiologic diagnosis in patients with end-stage cardiomyopathy can be challenging. In these cases, accurate diagnosis of patients before transplantation is critical and has priority to heart transplantation. Accurate diagnosis of patients before transplantation lead to proper treatment and subsequently slow down disease progression and improve patient outcome in particular diseases including sarcoidosis, myocarditis, iron toxicity-associated cardiomyopathy, and others. Moreover, it is important to accurately diagnose patients with diseases such as sarcoidosis, amyloidosis, and particular types of myocarditis due to the recurrence in the transplanted heart [10]. Although some complications of right ventricle endomyocardial biopsy including pneumothorax, air embolism, atrial arrhythmias, transient nerve palsies and paralysis, cardiac perforation, and tamponade have been reported [21], but if be performed by experienced operators, complication rates is very low [22]. It should be also noted that the use of endomyocardial biopsy particularly when a non-ischemic cardiomyopathy or myocarditis is suspected has a considerable impact on accurate diagnosis and appropriate treatment [10].

It is logical to expect that endomyocardial biopsy cannot be performed in early steps of cardiomyopathy diagnosis, but some convincing data exist which strongly support the role of endomyocardial biopsy in providing additive information besides echocardiography in a definite diagnosis of cardiomyopathy [22]. Diagnosis of cardiac diseases by imaging methods like echocardiography is non-invasive but some pathologic conditions like myocarditis and infiltrative cardiomyopathies often require to endomyocardial biopsy for definite diagnosis [23]. In fact, only endomyocardial biopsy, but not echocardiography, is capable to establish the nature of the etiological agents that are at a cellular level including toxic, infectious-inflammatory, infiltrative, or autoimmune processes. As mentioned before, in patients who are heart transplantation recipients, the histology of endomyocardial biopsy helps monitor allograft rejection grading. Furthermore, recently, molecular biology technology has identified more detailed information by endomyocardial biopsy sample. For example, immunohistochemical and viral genome assessment on endomyocardial biopsy provides a definitive etiological diagnosis at cellular and molecular levels that conduce to more specific treatment such as antiviral or immunosuppressive therapy [22]. The study had some limitations; as our center was one of the first approved hospitals in the country for cardiac transplant, most of the cardiac transplant candidates were referred across the country to our center and many are in an advanced and terminal stage of heart failure and sometimes hospitalized in CCU/ICU. Most of them did not perform all sophisticated tests and advanced imaging tools like CMR or endomyocardial biopsy or genetic assay due to clinical, financial, or logistical issues.

## Conclusions

In summary, based on the findings of the present study, it could be concluded that there is a significant discrepancy between echocardiography and pathology in the diagnosis of cardiomyopathy; therefore, it is required to accurate diagnosis of cardiomyopathy with new advanced cardiac imaging like CMR and detailed genetic and serologic assays and pathologic assessment to reach an appropriate treatment strategy. Additional robust studies in different populations with larger sample sizes are essential to confirm these data.

## Data Availability

The data used in this study are available from the corresponding author upon a reasonable request.

## Abbreviations

HT: Heart transplantation
HF: Heart failure
CMP: Cardiomyopathy
IDC: Idiopathic dilated cardiomyopathy
CT scan: Computed tomography scan
CMR: Cardiac magnetic resonance imaging
SPECT: Single photon emission computed tomography
EMB: Endomyocardial biopsy
ICD: Implantable cardioverter defibrillator
CRT: Cardiac resynchronization therapy
CABG: Coronary artery bypass graft
PCI: Percutaneous coronary intervention
CCU, ICU: Cardiac care unit, intensive care unit
LBBB: Left bundle branch
ASE: American Society of Echocardiography
